# Prescription Habits Related to Chronic Pathologies of Elderly People in Primary Care in the Western Part of Romania: Current Practices, International Recommendations, and Future Perspectives Regarding the Overuse and Misuse of Medicines

**DOI:** 10.3390/ijerph18137043

**Published:** 2021-07-01

**Authors:** Valentina Buda, Andreea Prelipcean, Carmen Cristescu, Alexandru Roja, Olivia Dalleur, Minodora Andor, Corina Danciu, Adriana Ledeti, Cristina Adriana Dehelean, Octavian Cretu

**Affiliations:** 1 Faculty of Pharmacy, “Victor Babes” University of Medicine and Pharmacy, Eftimie Murgu Square, No. 2, 300041 Timisoara, Romania; andreea.preli@yahoo.com (A.P.); carmencristescu@umft.ro (C.C.); corina.danciu@umft.ro (C.D.); afulias@umft.ro (A.L.); cadehelean@umft.ro (C.A.D.); 2 Research Centre for Pharmaco-Toxicological Evaluation, “Victor Babes” University of Medicine and Pharmacy, Eftimie Murgu Square, No. 2, 300041 Timisoara, Romania; 3 Faculty of Economics and Business Administration, West University of Timisoara, Vasile Parvan Boulevard, No.4, 300223 Timisoara, Romania; alexandru.roja@e-uvt.ro; 4 Clinical Pharmacy Research Group, Louvain Drug Research Institute, Universite Catholique de Louvain, E. Mounier Street, No. 81, 1200 Woluwe-Saint-Lambert, Belgium; olivia.dalleur@uclouvain.be; 5 Faculty of Medicine, “Victor Babes” University of Medicine and Pharmacy Eftimie Murgu Square, No. 2, 300041 Timisoara, Romania; andorminodora@gmail.com (M.A.); cretu.octavian@umft.ro (O.C.); 6 Advanced Instrumental Screening Center, “Victor Babes” University of Medicine and Pharmacy, Eftimie Murgu Square, No. 2, 300041 Timisoara, Romania

**Keywords:** aged people, primary health care, STOPP/START, Beers criteria, medical prescriptions for chronic pathologies, inappropriate prescribing

## Abstract

The European Commission’s 2019 report regarding the state of health profiles highlighted the fact that Romania is among the countries with the lowest life expectancy in the European Union. Therefore, the objectives of the present study were to assess the current prescription habits of general physicians in Romania related to medicines taken by the elderly population for chronic conditions in both urban and rural setting and to discuss/compare these practices with the current international recommendations for the elderly (American—Beers 2019 criteria and European—STOPP/START v.2, 2015 criteria). A total of 2790 electronic prescriptions for chronic pathologies collected from 18 community pharmacies in the western part of Romania (urban and rural zones) were included. All medicines had been prescribed by general physicians. We identified the following situations of medicine overuse: 15% of the analyzed prescriptions involved the use of nonsteroidal anti-inflammatory drugs (NSAIDs) for >2 weeks, 12% involved the use of a proton-pump inhibitor (PPI) for >8 weeks, theophylline was the bronchodilator used as a monotherapy in 3.17% of chronic obstructive pulmonary disease cases, and zopiclone was the hypnotic drug of choice for 2.31% of cases. Regarding the misuse of medicines, 2.33% of analyzed prescriptions contained an angiotensin-converting enzyme (ACE) inhibitor and an angiotensin II receptor blocker (ARB) for patients with renal failure in addition to vitamin K antagonists (AVKs) and NSAIDs in 0.43% of cases. Prescriptions for COX2 NSAIDs for periods longer than 2 weeks for patients with cardiovascular disorders accounted for 1.33% of prescriptions, and trihexyphenidyl was used as a monotherapy for patients with Parkinson’s disease in 0.18% of cases. From the included medical prescriptions, 32.40% (the major percent of 2383 prescriptions) had two potentially inappropriate medications (PIMs). Rural zones were found to be risk factor for PIMs. Decreasing the chronic prescription of NSAIDs and PPIs, discontinuing the use of hypnotic drugs, and avoiding potentially harmful drug–drug associations will have long term beneficial effects for Romanian elderly patients.

## 1. Introduction

The European Commission’s 2019 report regarding the state of health profiles highlighted the fact that Romania is among the countries with the lowest life expectancy in the European Union (EU) (75.3 years in Romania versus 80.9 years in the EU), with large discrepancies between individuals of different genders and education levels [1]. Women live for an average of 7 years longer than men, and the most educated men (with at least a tertiary education level completed) are expected to live 10 years longer than the least educated (who have not completed secondary education) [2].

Some of the causes of increased mortality involve behavioral risk factors (smoking, obesity, alcohol consumption, low physical activity, and poor nutrition in the form of excessive consumption of salt and sugar and low intake of fruits and vegetables), having a lower number of doctors and nurses per inhabitant, and having much lower health care costs compared with other EU countries (both per patient—1029 versus 2884 EUR/patient in the EU and per percent of gross domestic product (GDP)—5 versus 9.8% in the EU) [1].

It seems that the main causes of death are preventable and treatable pathologies, with diseases of the circulatory system (ischemic heart diseases and stroke) being the primary cause (58.2%), followed by cancer (lung, breast, or colorectal) and respiratory diseases [3].

In light of the current severe acute respiratory syndrome coronavirus (SARS-CoV-2) pandemic, we also have to highlight the susceptibility of elderly people to this virus [4]. Elderly people are more likely to develop a severe and critical form of the disease. Diseases such as cardiovascular diseases, acute respiratory distress syndrome, chronic obstructive pulmonary disease, and diabetes can predict poorer outcomes [5], putting elderly people at risk of faster clinical deterioration [6]. In Romania, The National Institute of Public Health provides a weekly report concerning the COVID-19 situation. Their statistics highlight that the median age of death due to COVID-19 is 71 years and that all people who have died from this disease have had at least one comorbidity. It has also been shown that 59.8% of deaths occurred in males [7]. In a case study, the European Commission’s H2020 Expert Group pointed that sex and gender can impact the outcome of contracting COVID-19 and that more men than woman die from acute infection [8]. Regarding the COVID-19 mortality rate in other European countries, data published at the beginning of February 2021 by the WHO Coronavirus Disease Dashboard showed the following: 3.51% in Hungary, 3.46% in Italy, 2.73% in Germany, 2.54% in Romania, 2.41% in France, and 2.09% in Spain (number of deaths/total number of reported cases) [9].

Primary care services in Romania seem to be less often used than hospital emergency services. Emergency services are often used for less urgent cases, thus increasing the inpatient care costs for the Romanian population [1]. Moreover, the vaccination rate is lower than the EU average (8% in Romania versus 44% in the EU among the elderly for influenza in 2017) [1,10].

Several studies have stated that inappropriate prescription is a major health issue among the elderly in all clinical settings [11,12,13]. The physiological changes that occur during the aging process, result in changes in the pharmacokinetics and pharmacodynamics of administered medicines. Moreover, the presence of comorbidities and polypharmacy, lead to negative outcomes regarding patient safety, such as adverse drug reactions (ADRs). These ADRs will later increase the prevalence and incidence of morbidity and mortality in geriatric patients [14].

In order to counteract this problem, clear rules and recommendations for proper utilization of medicines in the elderly population are required. Explicit criteria have been developed in order to improve the selection, efficiency, and safety of medication as well as the quality of health care services [15]. The first criteria that were developed were the Beers criteria, which were published in 1991 in the USA. They were later adapted and improved for European countries (due to several differences regarding approved medicines in the European market and treatment strategies), giving rise to the Screening Tool of Older Persons’ Prescriptions (STOPP) and the Screening Tool to Alert to Right Treatment (START) criteria, which were published in 2008. The American Geriatrics Society updates the Beers criteria every three years (starting from 2012), while the STOPP/START version 1 criteria were updated seven years after first being published, in 2015 [15]. The STOPP/START criteria are now recognized by several institutes and geriatric societies and are used by many European countries in routine clinical practice [16,17].

In 2016, in a population of elderly community members, Wallace et al. showed that when a minimum of two potentially inappropriate medicines were prescribed, the risk of an ADR was increased (according to the STOPP/START criteria). This, in turn, decreased patients’ quality of life and increased hospitalization rates over a follow-up period of 2 years [11]. Moreover, in 2016, Wauters et al. concluded that mortality and hospitalization rates are related to inappropriate medication prescription practices, such as overuse (prescribing more medicines than are clinically needed and with potential harmful effects that exceed the potential benefits) and misuse (incorrectly prescribing a medicine) of medicines [13,18,19,20].

Romania is currently lacking studies that demonstrate the problems in the healthcare system or in real-life situations (lack of current statistical data). Few studies (three in the last 9 years) have assessed the prescription appropriateness in the elderly population [14,21,22]. Moreover, the county and the city hospitals in Romania lack geriatric doctors, and the European guidelines for appropriate prescription in elderly people have not been implemented (and few specialists know about them) [23].

Early detection of frailty and intervention for elderly Romanians living independently (able to take care of themselves without being dependent on another human) must be a priority, as more than 65% of subjects, particularly women (divorced or widowed, with a higher risk, aged >75 years old), were considered frail in a study performed by Pislaru et al. in 2016 [24,25].

Therefore, the objectives of the present study were to assess the current prescription habits of general physicians in Romania regarding medicines taken as chronic treatments by the elderly population (in both rural and urban setting) and to discuss/compare our findings with the current international (USA—Beers 2019 Criteria and European—STOPP/START v.2, 2015) recommendations for the elderly [26,27].

## 2. Materials and Methods

### 2.1. Study Design, Setting, and Data Collection

This cross-sectional study included a total of 2790 electronic medical prescriptions for chronic pathologies collected from 18 community pharmacies in the western part of Romania (urban and rural zones) between January 2018 and June 2019, all written by general physicians. Regarding the Romanian classification of urban and rural zone, it is worth mentioning that some of the minimum indicators mentioned by Romanian legislation for urban zones are as follows: 5000 inhabitants/locality, 75% of the total employed population working in non-agricultural activities, 70% of all homes equipped with water supply installations, 55% of all homes equipped with a bathroom and toilet inside the house, seven hospital beds/1000 inhabitants, and 1.8 doctors/1000 inhabitants [28,29]. By chronic pathologies, we understand a human health condition/disease as lasting more than 3 months (long duration and slow progression), that cannot be prevented by vaccines or cured by medication [30].

**Figure 1:**
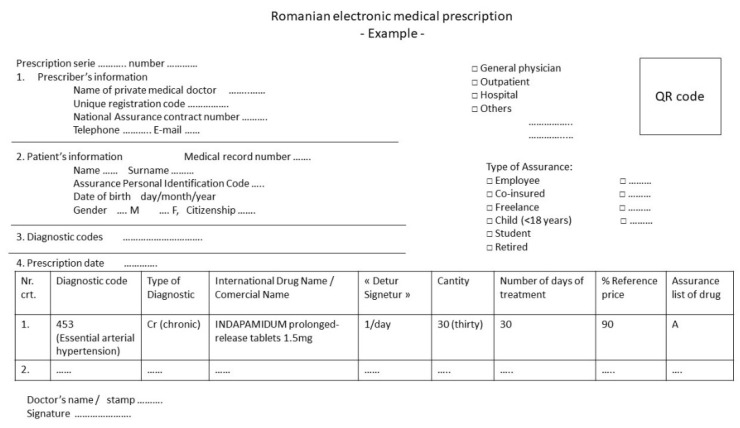
Example of a Romanian electronic medical prescription [31].

In Romania, electronic prescriptions for chronic pathologies can be issued over a period of 30, 60, or 90 days and contain a maximum of seven prescribed medicines (usually written using international drug names), which are reimbursed by the national health insurance system (Figure 1) [31].

When medical electronic prescriptions were collected (as the printed version) from community pharmacies (by the “pharmacy group”: pharmacist, 2 clinical pharmacist students, and 2 clinical pharmacists), the names and assurance personal identification codes of patients had been already blurred.

#### 2.1.1. Inclusion Criteria

Electronic prescriptions were included in this study based on age (≥65 years old), prescriber (general physician), and ambulatory treatment and duration (chronic treatment).

#### 2.1.2. Exclusion Criteria

Prescriptions did not meet the criteria for inclusion in this study if they were duplicates issued for the same patient but in different months (based on the patient’s gender, date of birth, prescriber, and medical record number of the patient, using Microsoft Excel). These were excluded by the “pharmacy group” that analyzed the prescriptions. Psychotropic and narcotic medications, such as benzodiazepines, barbiturates, opioids, and zolpidem (and not zopiclone), were also excluded from this study, as Romanian legislation (Law 339/2005) requires different prescription forms that are non-electronic and more secure for these types of medicines [32]. Moreover, over-the-counter (OTC) medicines and food supplements were also excluded, as they are not reimbursed by the national health insurance system and cannot be prescribed via electronic form.

### 2.2. Data Evaluation

The collected prescriptions were analyzed in face-to-face meetings by an interdisciplinary team of 10 specialists (cardiologist, psychiatrist, gastroenterologist, pulmonologist, generalist, pharmacist, two clinical pharmacists, and two clinical pharmacy students) over a period of 6 months, based on the 2019 Beers criteria and the STOPP/START v.2 criteria [26,27,33]. The meetings were scheduled once per week, and each session lasted for a minimum of 2 h.

**Figure 2:**
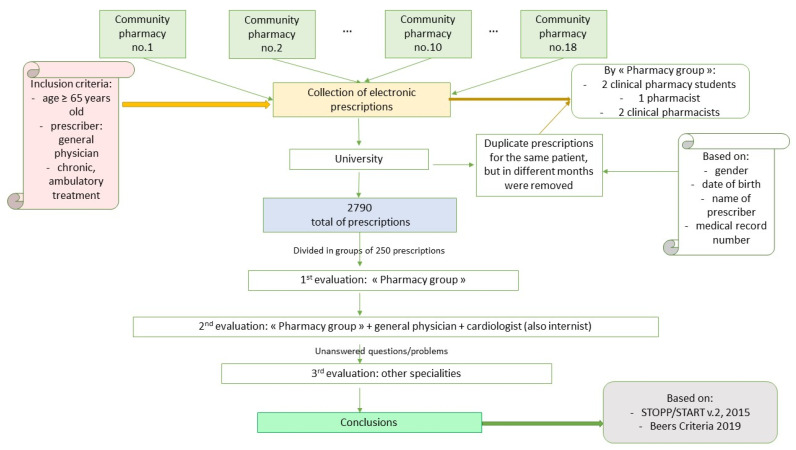
Schematic representation of the study’s methodology.

First, the collected medical prescriptions were divided in blocks of 250 prescriptions. Each block of medical prescriptions underwent a three-round screening evaluation: first, they were evaluated by the “pharmacy group”, then by the general physician and the cardiologist/internist (double specialization), and finally, unanswered questions/problems were managed with the help of other specialists (Figure 2). Final decisions were made based on full agreement by everyone using the Beers 2019 and STOPP/START v.2, 2015 criteria [26,27]. It is worth mentioning that the results of the first review performed by the “pharmacy group” were shared with the first physician reviewers (general physician and the internist).

Patients’ data were collected using chronic electronic prescriptions, and we did not have access to clinical data. We were able to identify the prescriber (e.g., general physician), the type of treatment (e.g., ambulatory and chronic), and the patients’ genders and ages (Figure 1).

Based on the diagnostic codes (attributed by the national health insurance system for each chronic pathology) of each prescription, we identified the main chronic conditions experienced by the patients.

Treatment duration was determined based on the days for which each prescription was issued (number of days of treatment, Figure 1). For the assessment of the duration of use of proton-pump inhibitors (PPIs) and H2 antagonists, only prescriptions issued for 60 or 90 days were counted in the final analysis.

Overall, 26 STOPP v.2, 2015 criteria were applied to the dataset [27]. Regarding the 2019 Beers criteria, only 17 could be applied [26]. All the applied criteria are listed in Appendix A of the present article.

### 2.3. Statistical Analysis

Data are presented either as the mean ± standard deviation or as percentages. SPSS v.17 statistical software (SPSS Statistics for Windows, version 17.0. Chicago: SPSS Inc.) was used for the analysis. For the sample size calculation, we conducted a power analysis test using G*Power 3.1 software, with 80% power, a significance level of 0.05, and an effect size of 5.31% [34,35]. We determined the descriptive statistics for the numerical variables (means and standard deviations) and for the qualitative variables (absolute and relative frequencies). Logistic regression was applied in order to determine the association between the number of potentially inappropriate medications (PIMs) and zone (rural/urban setting), age, gender, number of chronic conditions and of medicines. Chi-squared test was applied for categorical type variables and Mann–Whitney U test for numerical variables that were not normally distributed [36,37].

All collected electronic prescriptions for chronic conditions are stored under lock and key at the “Victor Babes” University of Medicine and Pharmacy, along with the flash drive containing the electronic data.

## 3. Results

### 3.1. Characteristics of the Analyzed Prescriptions and the Studied Population

A total of 2790 electronic prescriptions for chronic conditions (for 2790 patients) were included, of which 53.69% were issued by urban general physicians. Of the total prescriptions, 60.64% were for female patients, and the mean age of patients was 74.54 ± 7.22 years old. The vast majority (78.70%) of included medical prescriptions were written for a period of 30 days, and the mean number of medications per prescription was four (Table 1).

Table 2 presents the average number of medicines per medical prescription based on age category and gender.

From Table 2, it can be observed that for a given age group, men were prescribed more medications than women, suggesting an increased morbidity rate in the male gender.

**Figure 3:**
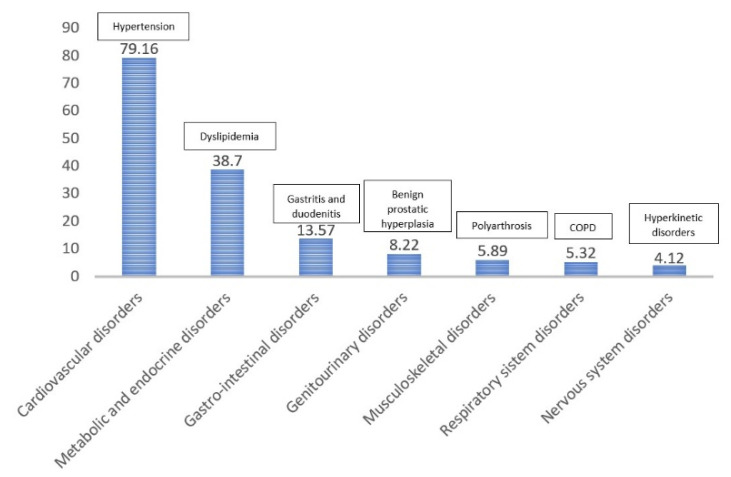
Most common chronic conditions requiring prescriptions.

Figure 3 presents the most common chronic conditions associated with the analyzed prescriptions based on the diagnostic code of each medical prescription. As expected, cardiovascular disorders were the most common chronic conditions encountered (around 79% of cases), followed by metabolic and endocrine disorders (38.7%), gastrointestinal disorders (13.57% of cases), respiratory system disorders, and genitourinary, musculoskeletal, and nervous system disorders (Figure 3).

### 3.2. Inappropriate Prescription Problems

Table 3 presents the problems associated with the overuse of medicines in elderly Romanian patients.

The prescription of NSAIDs and PPIs was the main problem identified regarding treatment duration. Of the included prescriptions, 15% had an NSAID prescribed for more than 2 weeks, and 12% had an PPI prescribed for more than 8 weeks (Table 3).

Moreover, theophylline was the bronchodilator used as a monotherapy in 3.17% of cases for patients with chronic obstructive pulmonary disease, while zopiclone was the hypnotic medicine used in 2.31% of cases (Table 3).

We also identified duplications of pharmacological class in 2.2% of medical prescriptions (Table 3).

Table 4 presents the identified problems associated with the misuse of medicines in Romanian elderly patients.

Concerning the misuse of medications, the most commonly encountered problems were drug–drug interactions, the prescription of COX2 NSAIDs for longer than 2 weeks for patients with cardiovascular disorders (1.33% of cases), and the prescription of trihexyphenidyl as a monotherapy for patients with Parkinson’s disorder (Table 4).

Regarding drug–drug interactions, 2.33% of analyzed prescriptions contained the association of an ACE inhibitor and an ARB for patients with renal failure (identified based on the diagnostic code), followed by the association of AVK and NSAIDs in 0.43% of cases (Table 4).

The percent of medical prescriptions in function of the number of potentially inappropriate medication (PIM) is presented in Table 5. In addition, 2383 medical prescriptions (85.41%) of total cases had a least one PIM.

It can be noticed that the highest percent of potentially inappropriate prescriptions (32.40%) included two potentially inappropriate medications (Table 5), from a total of 2383 prescriptions with PIM.

As presented in Table 6, urban zones were found to be protective factors for PIMs (OR = 0.582, with 95% CI = [0.482, 0.702], as well as a higher duration of treatment (OR = 0.995, with 95% CI = [0.991, 0.999]).

Rural zones were found to be risk factor for PIMs (Chi-squared test, *p* < 0.001, OR = 2.109, with 95% CI = [1.769, 2.516]) (Table 5 and Table 6).

## 4. Discussion

To the best of our knowledge, this is the first study to assess the prescription habits of general physicians for medications taken as chronic treatments by elderly patients in both urban and rural settings of the western part of Romania and to compare these with the international recommendations for aged people (USA—Beers 2019 criteria and European—STOPP/START v.2, 2015 criteria) [26,27].

This retrospective study, which included a large number of electronic prescriptions for chronic conditions for elderly patients prescribed by Romanian general physicians, showed that more than 85% had medication prescription problems. Below, we discuss some of the most frequent inappropriate prescriptions in light of current recommendations.

### 4.1. NSAIDs

The most commonly encountered problem in our study was the prescription of NSAIDs and therefore the overuse of this class of medicines by Romanian general physicians for the elderly population. The Beers 2019 criteria state that the administration of NSAIDs increases the risk of gastrointestinal bleeding and peptic ulcer development, especially if they are used as a chronic treatment for more than 1 year. The use of PPIs or misoprostol reduces, but does not eliminate, this risk. Moreover, the use of NSAIDs can increase blood pressure and induce kidney injury, which can aggravate heart failure, as they promote fluid retention and can increase mortality [26]. Therefore, it is recommended to use NSAIDs with caution in the lowest effective dose and for the shortest possible period of treatment (acute treatment), as they can induce several gastrointestinal, renal, and/or cardiovascular side effects, as described in Table 7 [40].

If these medicines must be used in the elderly, monitoring for common side effects is recommended, especially as several studies have shown that long periods of NSAID exposure increase the risk of acute kidney injury or chronic kidney disease progression, especially if NSAIDs are combined with certain other classes of medicines, such as ACE inhibitors/ARBs and/or diuretics [41,42,43]. We identified that 2% of cases (56 patients) involved the “triple whammy therapy” (association of RAAS inhibitor + diuretic + NSAID), and 1.15% (32 patients) involved the association of an ACE inhibitor/ARB + NSAID. Several studies have shown that “triple whammy” therapy increases the risk of acute kidney injury, with higher hospitalization rates, especially for men [44,45]. Moreover, the American Geriatric Society noted that NSAID use must be avoided in all patients with end-stage renal failure (CrCl <30 mL/min) [26].

It is important to take the risk of hyponatremia into consideration, as it is the most common electrolyte disorder encountered in clinical practice, especially in the elderly population (due to dehydration, polypharmacy, and comorbidities, which all can induce an electrolyte imbalance) [46]. All NSAIDs can induce hyponatremia, even if they are taken for only a few days, as they inhibit the action of the antidiuretic hormone (due to the reduction of renal prostaglandins), causing also water retention. Thus, physicians must consider this risk, as well as the associations of medicines that can aggravate hyponatremia, as even mild forms of hyponatremia are associated with negative clinical outcomes, such as cognitive impairment, falls, hospitalizations, and mortality [46]. Moreover, a higher mortality rate has been observed in patients with moderate or severe hyponatremia [47,48]. In addition, the STOPP/START v.2 criteria state that the use of selective serotonin reuptake inhibitor (SSRI) antidepressants (the most commonly prescribed medicines nowadays) must be avoided in patients with hyponatremia, as they can exacerbate this condition, while the 2019 Beers criteria also include tramadol in the list of medicines that can aggravate hyponatremia or cause inappropriate antidiuretic hormone secretion (SIADH) [26,27,48]. Thus, it is extremely important to decrease the number of chronic prescriptions of NSAIDs in the Romanian elderly population and to monitor sodium levels in patients undergoing chronic treatment with NSAIDs, as deleterious effects can arise, with extremely dangerous consequences.

In addition to prescriptions, our research group showed (in another study performed) that more than 65% of the Romanian population use NSAIDs for self-medication, especially patients with cardiovascular pathologies, despite the European Medicine Agency recommendations (on safety precautions of particular NSAIDs) and probably because diclofenac formulations are the cheapest medicines on the market [49]. Thus, members of the Romanian population are large consumers of NSAIDs, although Romania is among the EU countries with the highest incidence of acute coronary syndrome [1].

In the elderly, drug-induced nephrotoxicity is a frequent adverse reaction, which can precipitate acute or chronic diseases as well as increase morbidity and mortality. It has been shown to be responsible for 66% cases of renal failure in the elderly population [50]. Moreover, as renal function is affected by senescence, practitioners should avoid prescribing medicines that can increase this risk (for the elderly) when taken in association, as the renal blood flow, number of functional nephrons, and renal filtration rate are already affected by the aging process. Certain prescription associations also increase the risk of community-acquired hyperkalemia in the elderly [44,45].

Furthermore, we also identified associations of NSAIDs and AVKs in chronic electronic prescriptions. It is well known that this kind of association highly increases the hemorrhagic risk. Based on the fact that a very small proportion of the Romanian population has targeted INR (International Normalized Ratio) values under anticoagulant treatment, it is advisable to avoid this association for members of the Romanian elderly population [51].

### 4.2. PPIs

The second most frequently encountered problem was the prescription of PPIs for a period of more than 2 months. The 2019 Beers criteria mention the risks of bone loss, fracture, and *C. difficile* infection with long-term PPI treatment. Moreover, several publications have shown that long-term use (>2 months) of PPIs is associated with the following side effects, which are particularly harmful in elderly patients: vitamin B_12_ and iron deficiency, hypomagnesaemia, bone demineralization and fragility, intestinal and other infections (bacterial overgrowth, non-typhoidal *Salmonella*, *Campylobacter*, *Clostridium difficile*, community-acquired pneumonia), impaired cognition and affect, and increased risk of chronic kidney disease [52,53,54]. PPI use was recently associated with the onset of dementia and depression in the elderly population, although the exact mechanism by which this occurs is not clear (it might be due to vitamin B_12_ malabsorption). Thus, it is necessary to evaluate the risks and benefits when prescribing PPIs for the elderly for long periods of time and to consider potential drug–drug interactions in patients with polypharmacy, as PPIs are inhibitors of the P450 cytochrome [55,56].

### 4.3. Theophylline

We also identified the prescription of theophylline as a monotherapy by Romanian general physicians for patients with chronic obstructive pulmonary disease. Theophylline is a narrow therapeutic window bronchodilator. The 2019 Beers criteria and the STOPP/START v2 2015 criteria recommend the use of beta 2 adrenomimetic or anticholinergic bronchodilators, which are more effective and less toxic compared with theophylline, for the treatment of chronic obstructive pulmonary diseases [26,27,57].

Therapy with theophylline requires monitoring of its concentration in the serum, and patients can experience toxicity symptoms like arrhythmia or convulsions before nausea and vomiting occur (Table 8). Moreover, smokers require higher doses of the medication, and smoking cessation increases its toxicity. It is susceptible to drug–drug interactions with the following medicines: phenytoin, ciprofloxacin, co-trimoxazole, levothyroxine, and benzodiazepines [58]. In addition, as cardiovascular diseases are the primary pathologies encountered in the elderly Romanian population (based on our study findings using the percentages of chronic conditions and other European reports) [59], treatment with theophylline can increase the risk of cardiovascular complications with serious side effects, especially in patients with atrial fibrillation and congestive heart failure. Thus, due to the low risk/benefit ratio and despite its low price, it is recommended that theophylline is replaced with other bronchodilator medicines (β_2_-agonists or anticholinergics) with safer and more efficient profiles. Further, the 2019 Beers criteria mention an increased risk of theophylline toxicity when the drug is associated with other medicines, especially enzyme inhibitors (e.g., ciprofloxacin), administered for acute treatments [26].

### 4.4. Zopiclone

Regrettably, despite several recommendations not to treat habitual insomnia in the elderly with medications such as benzodiazepines and non-benzodiazepine receptor agonist hypnotics (Z drugs), these medications are still the most commonly prescribed drugs for this age group [61]. The 2019 Beers recommendations state that “Z drugs” produce adverse drug reactions similar to those of benzodiazepines, such as daily sedation, delirium, and increased risk of falls and fractures. Moreover, they induce minimal improvements in sleep latency and duration [26]. Romanian legislation (Law 39/2005) demands that benzodiazepine and zolpidem drugs are prescribed on secure prescription forms and not with the basic electronic formulary; this is why our study did not include these substances [32]. Only zopiclone can be prescribed with the classical electronic prescription form, and as expected, our study shows that it is overprescribed in the elderly population.

It is worth mentioning that older people are the most vulnerable and susceptible to developing side effects of sleep medication, Z drugs, and benzodiazepines, so it is recommended to use them at the lowest effective dose (half the adult dose) and for a short period of time (4 weeks) [61,62]. Moreover, the 2019 Beers criteria mention that they have minimal efficacy in treating insomnia, with a high probability of developing adverse reactions [26]. The current recommendations regarding insomnia treatment focus on cognitive behavioral therapy, as maintaining cognitive functioning is an important aspect that must be taken into account in the elderly [61]. Moreover, sleep-disordered breathing should be diagnosed in a timely manner and treated effectively in elderly patients, as it can be a prime cause of sleep disorders [61,62]. An alternative medicine for the treatment of sleep disorders could be melatonin (as its natural secretion decreases with age), which is better tolerated and has fewer side effects. Moreover, it was recently reported to have renal protective properties, which could be beneficial for elderly patients [50].

### 4.5. Misuse of Medicines

Regarding the main drug–drug interactions encountered, 2.33% of the prescriptions analyzed in this study involved the association of an ACE inhibitor and an ARB for patients with renal impairment. In 2017, the European Society of Cardiology recommended that this association should be avoided, if possible, because of the unclear results from clinical trials regarding its benefits and the higher risk of acute functional renal failure [63].

It is recommended that COX2 NSAIDs to be avoided as chronic treatments for patients with cardiovascular pathologies due to the high incidence of acute coronary complications (e.g., myocardial infarction) [27]. Moreover, the 2019 Beers recommendations state that COX2 NSAIDs and thiazolidinediones should be used with caution in patients with asymptomatic heart failure and avoided in those with symptomatic heart failure [26]. In the present study, we identified several prescriptions containing COX2 NSAID use for more than 2 weeks.

### 4.6. Study Limitations

As a study limitation, we must mention the fact that only electronic prescriptions for chronic conditions were included. A clear picture of the entire patient treatment regimen (acute, chronic, OTC drugs, and food supplements) would have provided more information, along with other clinical patient data (e.g., hepatic/renal function, ionogram). Moreover, the assessment of oral anticoagulation therapy in patients with atrial fibrillation/heart failure was not possible, as there is no diagnostic code available from the assurance company for this pathology, and novel oral anticoagulants (NOACs) were not reimbursed by the company when the prescriptions analyzed in this study were written. Due to the limited availability of the included data, only part of the 2019 Beers and STOPP/START v.2, 2015 criteria could be applied [26,27]. Moreover, the present study included prescriptions from only the western part of Romania; thus, the present results cannot be generalized to the entire country. Additionally, patients’ frailty was not assessed.

### 4.7. Correlations with the Scientific Literature

Our study results are in accordance with the studies performed by Primejdie et al. in 2012 and 2016, which highlighted that NSAIDs, benzodiazepines, zopiclone, and zolpidem are the pharmaceutical substances most frequently associated with safety concerns in ambulatory as well as in institutionalized patients [21,22]. In a study performed in Spain in 2019, where the two versions of the Beers criteria (2012 and 2015) and the two versions of the STOPP criteria (v.1, 2008 and v.2, 2015) were applied, benzodiazepines, proton-pump inhibitors, peripheral alpha-1 blockers, and NSAIDs were among the most common potentially inappropriate medications found [64]. Another study performed in Brazil in very old hospitalized patients emphasized (after applying the 2019 Beers criteria) that polypharmacy occurs in approximately 84.6% of cases and that the most commonly encountered PIMs are metoclopramide, omeprazole, regular insulin, and haloperidol [65]. A study performed on South Korean geriatrics and published in 2018 showed that chlorpheniramine and amitriptyline were the most frequently prescribed PIMs (after applying the Beers Criteria) [66].

Other studies on the Romanian elderly population have shown that aged people living in villages have a significantly higher rate of prolonged hospitalization due to the lack of nearby hospitals [67] and the lack of specialists in small cities and rural areas [68].In addition to this data, in the present study, we found rural zones to be a risk factor for the incidence of PIM which could contribute to the risk of ADR and also to hospitalization rates, as other studies reported [11]. Moreover, a study performed by Simionescu et al. highlighted the fact that in order to reduce the mortality rate due to cardiovascular diseases, the amount of health care spending per person must be increased by the government [68].

Another study that evaluated patients’ adherence to antihypertensive therapy in urban family medical practices concluded that Romania needs further strategies and management strategy methods to increase patients’ adherence to treatment [69].

### 4.8. Purpose of Solutions

Regarding health expenditure in Romania compared to other European countries, it can easily be observed that some of the budget allocated to the health sector could be invested more strategically, with higher efficiency, especially for the prevention of diseases that generate high treatment costs (if they are not discovered in time). From the point of view of investing public budgets in health care, there are notable differences between Romania and other EU countries [70]. In the last five years, according to statistics provided by Eurostat and the OECD (Organization for Economic Co-operation and Development), Romania has allocated about 5% of its gross domestic product to health spending [70,71]. When compared to Switzerland, which invests about 12.5% of its GDP in health, or the European average, which is close to 7% of the GDP, it is clear that there is a major difference between Romania and other civilized countries in this regard. Additionally, of interest for our research is that, according to Eurostat statistics, regarding the destination of invested health budgets, in Romania, approximately 54.4% is spent on treating and rehabilitating patients, 27% is spent on equipment and goods needed in the medical process, and only 18% is spent on other expenses, including prevention. We compared this with Switzerland, which ranks first in the allocation of financial resources for other activities, including prevention and found that only half the budget of other countries is allocated for preventive medicine in Romania [70,71]. (The percentage allocated to preventive medicine from the state budget is about 1.8%, far below the European average.)

Another distinctive aspect noted in the OECD statistics is the way in which health budgets are formed at the level of the European Union and the distinct characteristics of Romania. Whereas in states with a high-performing health system, the share of government schemes and private financial instruments prevails, in Romania, the citizens’ contributions are the main source of funding for the health system. Almost 70% of Romanian health system costs come from citizen contributions, compared to Switzerland, where citizens contribute 40% and the state identifies other ways, including private ones, to ensure the stability of the national health budget [70,71].

Moreover, the use of technology, digital innovation, and digitalization as part of the national strategy can contribute significantly to making public health spending more efficient [72]. The use of electronic patient medical records is an immediate action that should be implemented in the Romanian health care system. From an economic point of view, the emergence of the eHealthcare field could greatly optimize the spending of budgets and ensure better prevention [73,74,75].

Teamwork between specialists (doctors, pharmacists, nurses) is also mandatory for a patient-centered approach with a lower incidence of iatrogenic events [76].

### 4.9. Practical Implications

The present study highlights the urgent need for appropriate pharmacological treatment in order to reduce the iatrogenic risk associated with renal injuries, cardiovascular complications, and electrolyte imbalances. The use of the Beers and STOPP/START criteria could also be beneficial as guides for appropriate treatment, along with clinical judgment and taking into account the specific characteristics of patients [26,27].

Moreover, we emphasize the urgent need to improve and correctly implement prevention strategies and effective treatment programs and to reinforce the proficiency/suitability of primary care, especially for elderly Romanians (who take the largest number of medicines), as the population is aging [77,78].

### 4.10. Purpose of Further Studies

Larger multi-centric studies are needed in order to get a correct overview of the current Romanian practices of prescribing, based on the entire medical record of the patients.

## 5. Conclusions

Several prescription problems have been identified in the Romanian primary care setting for patients with chronic pathologies. NSAIDs, PPIs, H2 antagonists, theophylline, and zopiclone were found to be among the medicines prescribed more often than clinically needed and that have potential harmful effects that exceed potential benefits. Additionally, duplication of pharmacological classes was observed. The association of RAAS inhibitors in patients with renal failure in addition to the utilization of COX2 NSAIDs were among the most commonly observed problems involving incorrect prescription.

Thus, decreasing the chronic prescription of NSAIDs and PPIs, discontinuing the use of hypnotic drugs, and avoiding potentially harmful drug–drug associations will have long term beneficial effects for Romanian elderly patients.

## Figures and Tables

**Table 1:** Main characteristics of the analyzed prescriptions and patients.

Number of Prescriptions	Zone of Prescriptions		Sex Distribution		Average Age of Patients (years)	Days of Treatment			Average Number of Medicines/prescriptions
2790	urban	53.69%	female	60.64%	74.54 ± 7.22	30 days	60 days	90 days	4.29 ± 1.60
	rural	46.30%	male	39.36%		78.70%	3.66%	17.64%	

**Table 2:** The average number of medicine/prescriptions based on age.

Age Category	Gender	% of Total Prescriptions	Average Number of Medications/Prescriptions
65–69 years old	female	23.38%	4.14
	male	11.01%	4.72
70–74 years old	female	13.84%	4.36
	male	12.45%	4.53
75–79 years old	female	9.96%	4.40
	male	5.46%	4.77
80–84 years old	female	11.00%	4.51
	male	5.45%	4.51
85–89 years old	female	5.57%	4.32
	male	3.78%	5.27
90–94 years old	female	1.14%	3.11
	male	0.74%	6.63
95–99 years old	female	0.70%	3.00
	male		

**Table 3:** Overuse of medicines according to STOPP/START v.2, 2015 and Beers 2019 Criteria [26,27].

Problem Encountered	Pharmacological Class/Medicine	% of Total Prescriptions	n of Patients	Recommendations for the Elderly
**a. Duration of treatment**	**NSAIDs (>2 weeks)**	15%	418	To be used with caution at the lowest effective dose and for the shortest period of time (acute treatment).
	[STOPP/STARRT v.2, 2015; Beers 2019]			Monitoring of side effects.
	**PPIs (>8 weeks)**	12%	335	To evaluate the risk/benefit ratio when prescribing for longer periods of time (>8 weeks).
	[STOPP/STARRT v.2, 2015; Beers 2019]			Use with caution in patients with polypharmacy (inhibitors of cytochrome P450).
	**H_2_ antagonist (>8 weeks)**	2%	56	To evaluate the risk/benefit ratio when prescribing for longer periods of time (>8 weeks).
	[STOPP/STARRT v.2, 2015]			Potential drug–drug interactions in patients with polypharmacy. [38,39]
**b. Treatment indication** (i) Bronchodilator used in monotherapy for chronic obstructive pulmonary disease(ii) Hypnotic drugs	**Theophylline**	3.17%	88	More effective and less toxic agents currently available (beta 2 adrenomimetic or anticholinergic bronchodilators).
	[STOPP/STARRT v.2, 2015]			
	**Zopiclone**	2.31%	64	Prefer other treatment options with safer profiles and better tolerance.
	[STOPP/STARRT v.2, 2015; Beers 2019]			If used, prescribe in the lowest effective dose (half of the adult dose) and for a maximum period of 4 weeks.
**c. Duplication of pharmacological class**	**Diuretics (loop or thiazide) Beta blockers Dihydropyridines NSAIDs ACE inhibitors H_1_ antagonists**	2.20%	61	Avoid using two medicines with the same mechanism of action. Minimal clinical benefits when duplicated.
	[STOPP/STARRT v.2, 2015]			Exacerbation of side effects.

Legend: NSAID, nonsteroidal anti-inflammatory drug; PPI, proton-pump inhibitor; ACE, angiotensin-converting enzyme; Screening Tool of Older Persons’ Prescriptions (STOPP); Screening Tool to Alert to Right Treatment (START).

**Table 4:** Misuse of medicines according to STOPP/START v.2, 2015 and Beers 2019 Criteria [26,27].

Problem Encountered	Pharmacological Class/Medicine	% of Total Prescriptions	n of Patients	Recommendations for the Elderly
a. Drug–drug interactions	**ACE inhibitors + ARBs**	2.33%	65	Avoid the association.
	[STOPP/STARRT v.2, 2015; Beers 2019]			High risk of hyperkalemia, renal injuries.
	**α_1_ blockers + Furosemide**	1.36%	38	Avoid the association.
	[STOPP/STARRT v.2, 2015; Beers 2019]			High risk of urinary incontinence.
	**AVK + NSAIDs**	0.43%	12	Avoid the association.
	[STOPP/STARRT v.2, 2015]			Major risk of gastro-intestinal bleeding.
	**Beta blockers + Verapamil/diltiazem**	0.33%	9	Avoid the association.
	[STOPP/STARRT v.2, 2015]			Cardiac depression, heart block.
	**Associations of CNS depressants**	0.18%	5	Avoid the association.
	[Beers 2019]			Central nervous system depression, with increased risk of falls and fractures.
b. Drug-pathology interactions	**Cardiovascular disorders + COX_2_ NSAIDs (>2 weeks)**	1.33%	37	Avoid the association.
	[STOPP/STARRT v.2, 2015; Beers 2019]			Increased risk of cardiovascular complications (stroke, myocardial infarction).
c. Drug class	**Trihexyphenidyl (monotherapy) for Parkinson’s disorder in patients** **≥65 years old**	0.18%	5	More effective substances currently available.
	[Beers 2019]			Increased risk of anticolinergic side effects.

Legend: ARB, angiotensin II receptor blocker; AVK, vitamin K antagonist; CNS, central nervous system.

**Table 5:** Percent of medical prescriptions with potentially inappropriate medication (PIM).

n of PIM/Prescription	% of Total Prescriptions with PIM	% of Rural Zones	% of Urban Zones
1	30.96	19.76	11.20
2	32.40	14.69	17.71
3	22.26	17.03	5.22
4	8.48	2.12	5.08
≥5	5.90	1.89	5.30

**Table 6:** Logistic regression considering PIM (Yes/No) as a dependent variable.

Variables in the Equation	B	S.E.	Wald	df	Sig.	Exp(B)	95% CI for EXP(B)	
							Lower	Upper
County	−0.659	0.101	42.125	1	0.000	0.518	0.424	0.631
Zone	−0.541	0.096	32.043	1	0.000	0.582	0.482	0.702
Gender	0.024	0.093	0.068	1	0.794	1.025	0.854	1.229
Age	0.005	0.006	0.732	1	0.392	1.005	0.993	1.017
n of medicines	−0.048	0.041	1.392	1	0.238	0.953	0.880	1.032
n of diagnostics	−0.003	0.053	0.004	1	0.950	0.997	0.899	1.105
Days of treatment	−0.005	0.002	5.878	1	0.015	0.995	0.991	0.999
Constant	1.044	0.513	4.139	1	0.042	2.840		

**Table 7:** Main side effects of NSAIDs [40].

Main Side Effects of NSAIDs		
Gastrointestinal	Cardiovascular	Renal
dyspepsia peptic ulcer gastrointestinal bleeding gastrointestinal perforation	edema hypertension myocardial infarction stroke congestive heart failure thrombotic events	sodium retention edema electrolyte imbalance reduction of glomerular filtration rate chronic kidney disease
Mechanism: inhibition of prostaglandin synthesis, which decreases the protective action of the gastrointestinal mucosa; fewer side effects with COX2 selective drugs but a higher cardiovascular risk.	Mechanism: inhibition of prostaglandin synthesis and elevation of serum aldosterone, which leads to hypertension and sodium retention.	Mechanism: inhibition of prostaglandin and thromboxane synthesis, which induces renal vasoconstriction, reduced renal perfusion, and impaired renal function.

**Table 8:** The most common side effects of theophylline [60].

Most Common Side Effects of Theophylline			
Neurological	Cardiovascular	Respiratory	Gastrointestinal
agitation irritability tremor hallucination insomnia	tachycardia atrial fibrillation hypotension cardiac arrest	tachypnea acute lung injury respiratory alkalosis	nausea vomiting abdominal pain

## Data Availability

The data presented in this study are available on request from the corresponding author.

## Appendix A

**STOPP/START v.2, 2015 Criteria Used** [27]:	**2019 Beers Criteria Used** [26]:
1. Prescribed medicine with no clinical indication 2. Prescribed medicine beyond the recommended period of administration. 3. The duplication of a drug class e.g., two concurrent NSAIDs, anticoagulants, ACE (angiotensin-converting enzyme) inhibitors, SSRIs (selective serotonin reuptake inhibitors), loop diuretics (before considering a new agent, an optimization of a single drug class should be taken into consideration). 4. The combination of a Beta-blocker with verapamil or diltiazem (risk of heart block). 5. The use of centrally-acting antihypertensive (e.g., methyldopa, clonidine, rilmenidine, moxonidine), unless lack of efficacy, or clear intolerance, with other classes of antihypertensive (older people tolerate them less well). 6. The combination of aldosterone antagonists (e.g., spironolactone, eplerenone) with concurrent potassium-conserving drugs (e.g., angiotensin receptor blocker’s, ACEI’s, amiloride, triamterene) without monitoring of serum potassium (risk of dangerous hyperkalaemia, i.e., >6.0 mmol/l – serum K should be monitored regularly, i.e., at least every 6 months). 7. The use of ACE inhibitors or Angiotensin Receptor Blockers in patients with hyperkalaemia. 8. The use of Loop diuretic as first-line treatment for hypertension (in regard to safety, other effective alternatives should be taken into consideration). 9. The use of loop diuretic as first line treatment for hypertension in patients associating urinary incontinence (may exacerbate incontinence). 10. Association of antiplatelet agents with direct thrombin inhibitor, vitamin K antagonist or factor Xa inhibitors in patients with cerebrovascular or peripheral arterial disease, stable coronary (the dual therapy did not bring any new benefits). 11. Association of NSAID with direct thrombin inhibitor, vitamin K antagonist or factor Xa inhibitors (risk of major gastrointestinal bleeding). 12. Association of NSAID with concurrent antiplatelet agent(s) without using the PPI as prophylaxis (increased risk of peptic ulcer disease). 13. The use of tricyclic antidepressants in patients with dementia, prostatism, cardiac conduction abnormalities, narrow angle glaucoma, or prior history of urinary retention (risk of worsening these conditions). 14. Initiation of tricyclic antidepressants as first-line antidepressant treatment (other are available with a lower risk of adverse drug reactions). 15. The use of neuroleptics with moderate-marked antimuscarinic/anticholinergic effects (chlorpromazine, clozapine, promazine flupenthixol, zuclopenthixol) in patients with history of prostatism or previous urinary retention (high risk of urinary retention). 16. The use of antipsychotics (i.e., other than clozapine or quetiapine) in patients with Parkinson disease or Lewy Body Disease (risk of severe extra-pyramidal symptoms). 17. The use of anticholinergics/antimuscarinics in patients with dementia or delirium (risk of exacerbation of cognitive impairment). 18. The use of neuroleptics as hypnotics, unless sleep disorder is due to psychosis or dementia (risk of hypotension, confusion, extra-pyramidal side effects, falls). 19. The use of PPI at full therapeutic dosage for >8 weeks for uncomplicated peptic ulcer disease or erosive peptic oesophagitis (it should be taken into consideration the reduction of dose or earlier discontinuation). 20. The use of theophylline for COPD as monotherapy (there are more effective and safer alternative; due to its narrow therapeutic index the risk of adverse effects is high). 21. The use of NSAID for a long-term (>3 months) for symptom relief of osteoarthritis pain where paracetamol has not been tried (simple analgesics preferable and usually as effective for pain relief). 22. The use of NSAID for a long-term or colchicine (>3 months) for chronic treatment of gout where xanthine-oxidase inhibitor (e.g., febuxostat, allopurinol) are not contraindicated. 23. Prescription of COX-2 selective NSAIDs in patients with concurrent cardiovascular disease (increased risk of stroke and myocardial infarction). 24. Prescription of neuroleptic drugs (may cause gait dyspraxia, Parkinsonism). 25. Prescription of Hypnotic Z-drugs, e.g., zolpidem, zopiclone, zaleplon (risk of fall, protracted day time sedation, ataxia). 26. The association of two or more drugs with antimuscarinic/anticholinergic effects (e.g., tricyclic antidepressants, tricyclic antidepressants, first generation antihistamines) (increased anticholinergic toxicity)	1. Antiparkinsonian gents (e.g., trihexyphenidyl)—more effective substances are currently available for the treatment of Parkinson disease; not recommended for the prevention/treatment of extrapyramidal symptoms with neuroleptics. 2. Peripheral alpha-1 blockers (e.g., doxazosin, prazosin) for treatment of hypertension—high risk of orthostatic hypotension in the elderly; more effective substances are currently available (superior risk/benefit ratio). 3. Central alpha-agonists—high risk of central nervous system side effects; risk of bradycardia and orthostatic hypotension if used for chronic treatment of hypertension. 4. Antidepressants (e.g., amitriptyline, doxepin > 6 mg/day, used alone or in combination)—intensive anticholinergic properties, risk of sedation and orthostatic hypotension. 5. Neuroleptic drugs used in patients with dementia – higher risk of stroke, cognitive decline, and death. 6. Benzodiazepine receptor agonist hypnotics (e.g., zopiclone)—side effects comparable to those of benzodiazepines in the elderly population. 7. Metoclopramide—risk of several side effects (e.g., extrapyramidal symptoms), especially with chronic treatment or in frail elderly patients. 8. Proton-pump inhibitors—risk of *Clostridium difficile infection* and bone loss or fracture. 9. Oral NSAIDs—increased risk of gastrointestinal bleeding or peptic ulcer development if administered as a chronic treatment or if associated with other medicines (e.g., corticosteroids, anticoagulants, antiplatelet agents). Higher risk of blood pressure augmentation and renal injuries. 10. Ketorolac and indomethacin (oral or parenteral use)—higher risk of gastrointestinal and renal problems. 11. Skeletal muscle relaxants (e.g., chlorzoxazone)—risk of anticholinergic effects, somnolence, fractures. 12. RAAS inhibitors (ACEIs, ARBs) or potassium-sparing diuretics associated with other RAAS inhibitors—risk of hyperkalemia. 13. Opioids associated with gabapentin/pregabalin—risk of severe sedation, fractures, respiratory depression, and death. 14. Association of two anticholinergic drugs—high risk of cognitive decline. 15. Association of three or more psychotropic drugs (e.g., antidepressants, antiepileptics, hypnotics, neuroleptics, opioids)—high risk of falls and fractures. 16. Corticosteroids (oral or parenteral)—increased risk of gastrointestinal complications. 17. Peripheral alpha-1 blockers associated with loop diuretics—high risk of urinary incontinence in elderly women.
Legend: NSAID, nonsteroidal anti-inflammatory drug; PPI, proton-pump inhibitor; ACE, angiotensin-converting enzyme; SSRIs, selective serotonin reuptake inhibitors; RAAS inhibitors, renin-angiotensin-aldosterone system inhibitors.	
